# Coexistence of gastric gastrointestinal stromal tumor, intro-abdominal and retroperitoneal liposarcomas –a case report

**DOI:** 10.1186/s12885-018-4886-4

**Published:** 2018-10-11

**Authors:** Yong Zhou, Xu-Dong Wu, Quan Shi, Chuan-hai Xu, Jing Jia

**Affiliations:** 1 Department of General surgery, Yancheng City No.1 people’s hospital, Yancheng, Jiangsu Province, 224005 China; 2 Department of Gastroenterology, Yancheng City No.1 people’s hospital, 16 Yuehe Road, Yancheng, Jiangsu Province, 224005 China; 3 Department of Radiology, Yancheng City No.1 people’s hospital, Yancheng, Jiangsu Province, 224005 China; 4 Department of Pathology, Yancheng City No.1 people’s hospital, Yancheng, Jiangsu Province, 224005 China; 5 Department of Nephrology, Yancheng City No.1 people’s hospital, Yancheng, Jiangsu Province, 224005 China

**Keywords:** GIST, Intro-abdominal liposarcoma, Retroperitoneal liposarcoma, Myxoid liposarcoma

## Abstract

**Background:**

Gastric gastrointestinal stromal tumor (GIST), intro-abdominal and retroperitoneal neoplasms are distinct tumors arising from different cell layers; therefore, coexistence of such tumors is relatively rare.

**Case presentation:**

A man complained of early satiety for 2 mouths, whose upper gastrointestinal (GI) endoscopy showed a tumor arising from the greater curvature of gastric body and extending into the lumen. Abdominal computed tomography (CT) revealed coexistence of gastric, intro-abdominal and retroperitoneal masses. Wedge resection for gastric tumor, resection for intro-abdominal and retroperitoneal tumors were done. The postoperative histological examination suggested simultaneous development of a gastric GIST, intro-abdominal and retroperitoneal myxoid liposarcomas.

**Conclusion:**

Although both GISTs and liposarcomas originate from mesenchymal tissues, simultaneous development of a gastric GIST, intro-abdominal and retroperitoneal liposarcomas is the first such case to be reported in the literature.

## Background

Past decades have witnessed frequent cases of synchronous occurrence of a GIST and another neoplasms [1, 2]. However, gastric GISTs simultaneous development of a liposarcoma is rarely reported. Liposarcoma represents 20–30% of adult soft tissue tumors, most commonly -arising from the extremities, followed by the retroperitoneum [3]. Furthermore, its abdominal localization occurs only in 5% of cases [3]. Although both GISTs and liposarcomas originate from mesenchymal tissues, simultaneous development of a gastric GIST, intro-abdominal and retroperitoneal liposarcomas is extremely rare in the literature.

## Case presentation

A 56-year-old male with early satiety for 2 mouths was admitted to our hospital. There was no history of weight loss, without relevant past and family history. An 18*25 cm oval tumor with medium texture was palpable below the left costal margin during the physical examination.

**Fig. 1:**
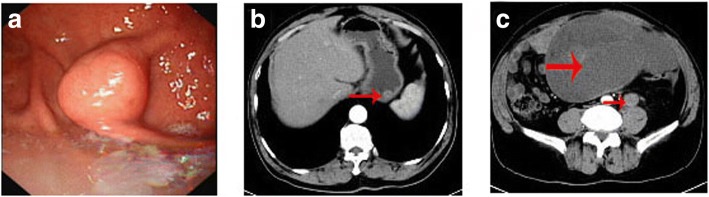
**a** Upper gastrointestinal (GI) endoscopy showed a tumor arising from the greater curvature of gastric body and extending into the lumen. **b** CT scan showed a marked enhancement of polypoid mass protruding into the gastric lumen. **c** CT scan obtained at a lower level displayed a huge, well-circumscribed, lobulated, intro-abdominal mass and a heterogeneous, round-like mass close to the left psoas

The routine biochemical and hematogical parameters were within normal limits, and tumour markers including CA-125, carcinoembryonic antigen (CEA) and CA19–9 levels were nothing special. Upper gastrointestinal endoscopy (GI) revealed a tumor arising from the greater curvature of gastric body and extending into the lumen (Fig. 1). Contrast enhanced CT scans of the abdomen showed a marked enhancement of polypoid mass protruding into the gastric lumen, a large poorly enhancing oval mass in the left abdomen and a heterogeneous round-like tumor adjacent to the left psoas (Fig. 1). Axial plain CT revealed the intro-abdominal tumor with CT values ranging from − 78 HU (consistent with fatty tissue) to 27 HU (related to the pancreas), with a mean CT value as 2 HU. However, the retroperitoneal tumor had CT values ranging from − 57 HU (consistent with fatty tissue) to 18 HU (related to the blood), with a mean CT value as 10 HU. The patient was diagnosed with coexistence of a gastric GIST, intro-abdominal and retroperitoneal tumors preoperatively.

A surgical operation was performed. Intraoperatively, a huge oval lobulated oozing soft mass (about 18.0*25.0*15.0 cm) originating from the descending colon mesentery was identified. A tumor (about 2.5*2.0*2.0 cm) at the greater curvature of gastric body and a tumor (about 5.0*4.0*2.5 cm) close to the left psoas were detected. Adjacent to the anterior wall of the abdominal aorta, the intro-abdominal tumor was surrounded by the small bowel and left hemicolon. The round-like lobulated retroperitoneal tumor was found among the left psoas, left iliac vessels, sigmoid colon and intro-abdominal tumor. The tumors were well-circumscribed from the surrounding organs and without signs of infiltrating tumor growth. Furthermore, the tumors were not related to the adjacent major vessels, without the detection of distant metastasis or nodal involvement. Wedge resection for gastric tumor, complete resection for intro-abdominal and retroperitoneal tumors were performed.

**Fig. 2:**
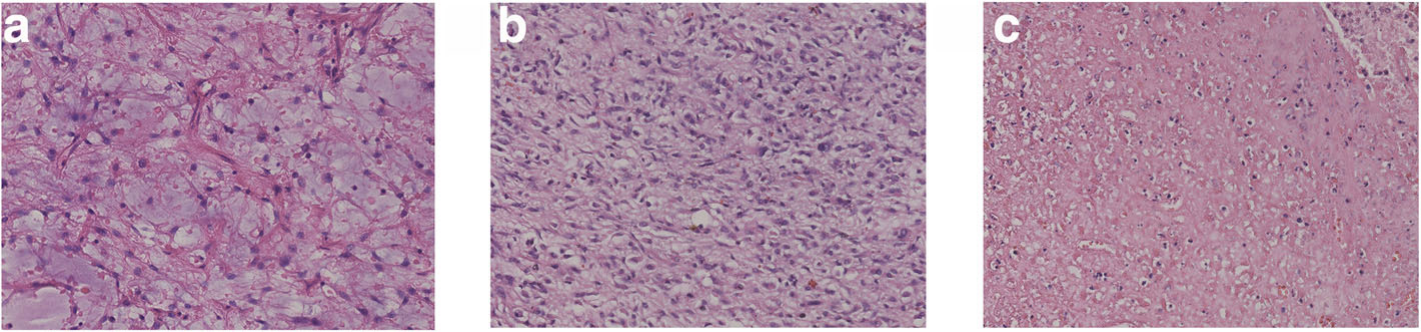
**a** Microscopic image of GIST (× 20 magnification). **b** Microscopic image of intro-abdominal liposarcoma (× 20 magnification). **c** Microscopic image of retroperitoneal liposarcoma (× 20 magnification)

In histopathological examination, the intro-abdominal tumor was a myxoid liposarcoma and the retroperitoneal mass shared the same pathological type with the tumor (Fig. 2).

**Fig. 3:**
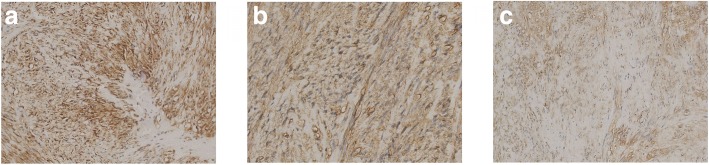
The immunohistochemistry indicated strong staining for c-Kit/CD117 (**a**, × 20 magnification), CD34 (**b**, × 20 magnification), Dog-1 (**c**, × 20 magnification)

Further histopathological examination of the gastric carcinoma indicated a GIST of the low-risk category (mitotic index < 5 mitoses/50 high-power fields) (Fig. 2). Immunohistochemistry displayed strong staining for c-Kit/CD117, Dog-1 and CD34, while expression of SMA and Desmin was negative (Fig. 3). Furthermore, mutations in KIT exons and PDGFRA exons were evaluated in the sample, but, nothing special was found.

**Fig. 4:**
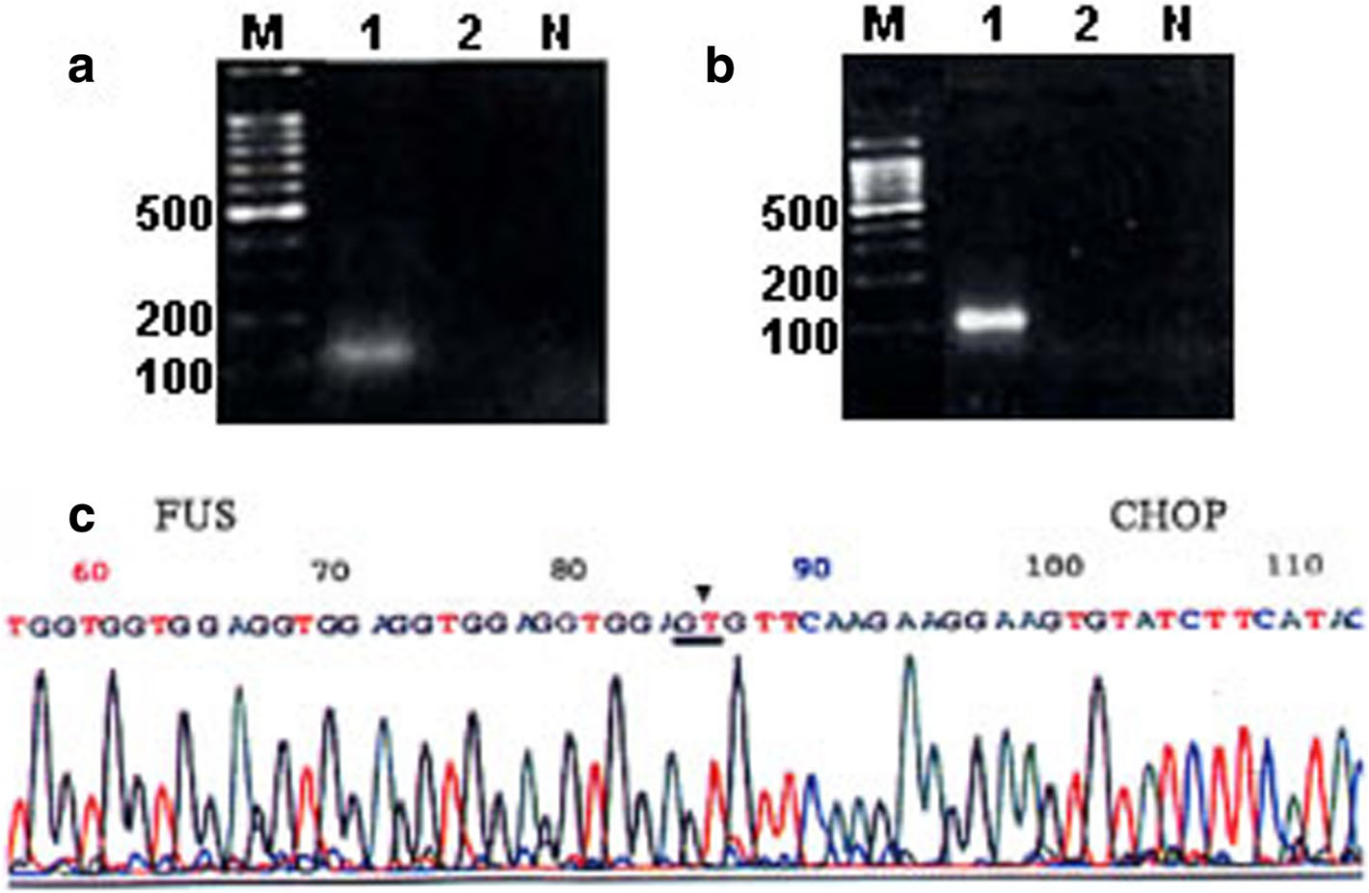
M: DNA marker (DL2000), Lane 1: retroperitoneal liposarcoma, Lane 2: intro-abdominal liposarcoma, N: Negative control, ▼: show the location of FUS-CHOP fusion. **a** Result of RT-PCR Assay of samples for β-actin mRNA. **b** Result of RT-PCR Assay of samples for FUS-CHOP mRNA. **c** Result of colonies sequencing for PCR products of FUS-CHOP mRNA

Nested reverse transcription-polymeras-ase chain reaction (RT-PCR) technique was employed to detect the FUS-CHOP mRNA expression in the formalin-fixed paraffin-embedded lipsarcoma samples. Type II FUS-CHOP mRNA was successfully detected in the retroperitoneal liposarcoma (Fig. 4). The postoperative course was uneventful. Adjuvant radiotherapy was targeted to the former liposarcoma bed: Dosage in total, 50 Gy; single dose, 1.6 Gy. The patient received CT scans twice a year, displaying no evidence of tumor recurrence in a follow up period of 15 months.

## Discussion

GISTs, comprising about 0.1–3% of all gastrointestinal malignant tumors, are rare.These tumors, strongly expressing the c-KIT protein (CD117; a type III tyrosine kinase receptor encoded by the c-kit proto-ongogene), are identified a**s** arise from interstitial cells of Cajal or their precursors [2]. As one of the most common mesenchymal neoplasms of the gastrointestinal tract, GISTs arise mainly from the stomach, small bowel and duodenum. Although GISTs usual present with alimentary tract hemorrhage, abdomen mass, abdominal pain and gastrointestinal obstruction, one-third of all cases are found incidentally. As shown in our case, based on the tumor detected during gastrointestinal endoscopy and CT scans due to the symptoms related to the huge liposarcoma, the gastric GIST could be classified as an incidental tumor.

According to the World Health Organization (WHO), lipomatous tumors were classified into well-differentiated, myxoid, pleomorphic and dedifferentiated subtypes [4]. Myxoid liposarcoma, a common subtype, is usually asymptomatic, incidentally identified, which occurs most commonly in deep soft tissues of the extremities while uncommon sites include the head and neck, thorax, and subcutis [5].The retroperitoneum is the most frequent site of liposarcoma. Up to 40% of liposarcomas occur in the intro-abdominal localization while those originated from the mesentery are rare. It is worth noting that primary retroperitoneal myxoid liposarcomas have been considered rare or even nonexistent [6], according to the recent studies. Furthermore, clinicopathologic studies confirmed that myxoid liposarcomas prefer to metastasize to deep soft tissues or bones, and the retroperitoneum stands for one of the most common metastatic locations [7]. As shown in our case, retroperitoneal liposarcoma could be considered as a metastatic mass from intro-abdominal myxoid liposarcoma.

Both GISTs and liposarcomas arise from mesenchymal tissues, coincidence of which is rarely reported. We only found two previously reported cases in the literature [8, 9]. One case showed a gastric GIST coexistence of a retroperitoneal liposarcoma [8], while the other reported a case of coincident abdominal carcinoid tumor, GIST and well-differentiated liposarcoma in a NF1 patient [9]. Various hypotheses including gene mutations, expression of metallothioneins (MT) and influenced neighboring tissues by the same carcinogen have been used to explain the simultaneous development of a GIST and other carcinomas [10, 11]. It is worth noting that genetic changes and polymorphisms play an important role in the development of sarcomas [12]. Furthermore, there is emerging data on genetic polymorphisms associated with susceptibility to a wide range of neoplasms [13, 14], which can be reasonably explained by genetic changes and polymorphisms.

Radical surgery is the main treatment for all mesenchymal tumors. Standing for the only curable chance for primary GIST, surgical resection can be achieved by only a wedge resection of the stomach or a segmental resection of the small bowel, while extensive surgery is occasionally performed for larger or poorly positioned GISTs [15]. Surgical resection is the only effective treatment for liposarcoma. During the procedure, the tissues or the organs adherent to the tumor usually need to be removed [16]. Even debulking surgery is sometimes helpful to treat with nettlesome symptoms of recurrence or un-resectable liposarcoma [17]. However, debulking surgery is not recommended in the recurrent or un-resectable GIST and for these patients, tyrosine-kinase inhibitor is a reasonable choice. In our case, the patient was diagnosed as simultaneous development of low grade gastric GIST, intro-abdominal andretroperitoneal myxoid liposarcomas. The evidence supporting the application of chemotherapy and radiotherapy for liposarcoma is limited [18]. A retrospective study showed a good local control rate for myxoid liposarcoma patients accepting surgery and radiotherapy [19], thereby, the patient was suggested to accept radiotherapy. The patient received CT scans twice a year, displaying no evidence of tumor recurrence in a follow up period of 15 months, making a long follow-up period necessary.

## Abbreviations

CEA: Carcinoembryonic antigen
CT: Computed tomography
GI: Gastrointestinal endoscopy
GIST: Gastrointestinal stromal tumor
MT: Metallothioneins
WHO: World Health Organization
